# Competency training: Using the ICO cataract rubric to learn and teach cataract surgery

**Published:** 2018-07-31

**Authors:** William Dean

**Affiliations:** 1 Clinical Research fellow: London School of Hygiene and Tropical Medicine, London, UK.

**Assessing surgical skills is a challenge. The ICO cataract rubric offers a helpful solution.**

**Figure:**
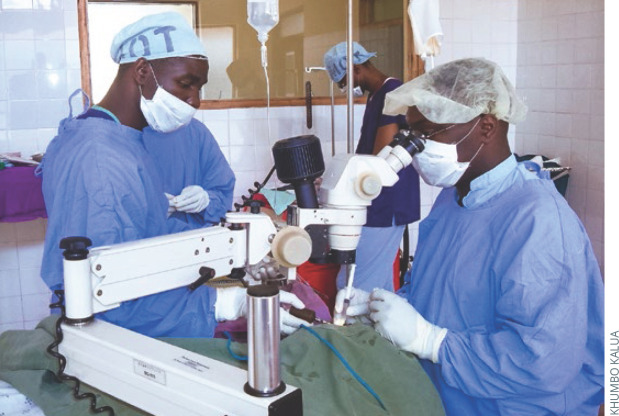
Surgical training may differ from one institution to another. MALAWI

Trainee eye surgeons learn surgical techniques in various ways. Often, they observe many operations, then start performing different stages of an operation under the supervision of a senior ophthalmologist. After further practice in either a wet lab (if available) or under supervision in the operating theatre, full surgical procedures, such as cataract surgery, are performed.

But practice is not enough. Before surgeons can qualify, their surgical technique must be assessed. Unfortunately, surgical skills are often the least well assessed component of clinical education and is often done subjectively, for example by evaluating a retrospective report from a supervisor. As a result, the standards surgeons achieve may differ from one training institution or supervisor to another.

## How can the assessment of surgical skills be improved?

**Figure 1:**
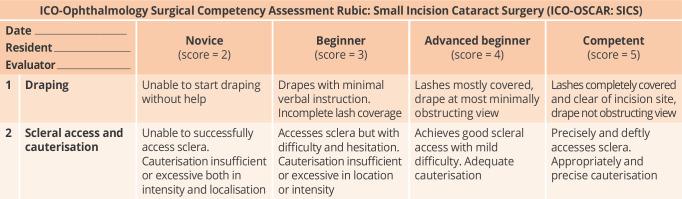
An example from the ICO-OSCAR for small-incision cataract surgery (SICS)

In order to ensure standard and robust assessment, the International Council of Ophthalmology (ICO) has created the Ophthalmology Surgical Competency Assessment Rubric, or ICO-OSCAR.^1^,^2^ ICO-OSCAR is known as a ‘rubric’; it breaks an operation down into its separate steps (e.g., from ‘draping’ to ‘wound closure’) and sets clear guidelines for the different levels of skill with which a step is performed (from ‘novice’ to ‘competent’). The steps of the operation are arranged in rows, and the columns correspond to the level of skill achieved (see Figure 1).

The ICO have developed many OSCARs for different procedures; including extracapsular cataract extraction, phacoemulsification, pediatric cataract surgery, small incision cataract surgery, strabismus, lateral tarsal strip surgery, trabeculectomy, and vitrectomy; all available in English. Selected ICO-OSCAR's are available in Mandarin Chinese, French, Portuguese, Russian, Spanish, and Vietnamese.^1^

The ICO-OSCAR also assesses more general aspects of surgical performance. These are termed the ‘global indices’ and include central eye positioning under the microscope, tissue handling, intraocular spatial awareness, and the overall fluidity of the procedure.

## Benefits for learning and teaching

The ICO-OSCARs provide a wonderful tool to learn, reflect, teach, and assess eye surgical performance.

As a **training tool**, it helps trainers to assess surgical skills in a structured and objective way. Knowing exactly what will be assessed makes it possible to plan the training programme in detail, so that trainees are clear about what is expected of them and have lots of opportunities to practice.

The ICO-OSCAR is also an exceptionally valuable **learning tool**, particularly if it is shared with trainees from the outset. It is a great learning exercise for them to study the OSCAR rubric and aim for ‘competent’ at each step. What is even more profoundly effective, is for a trainee cataract surgeon to video-record an operation they perform and assess or mark it themselves using the OSCAR rubric, and then reflect on what they need to improve. Such reflective learning is invaluable, especially when a trainer gives additional feedback.
